# Functional Assessment of Pluripotent and Mesenchymal Stem Cell Derived Secretome in Heart Disease

**Published:** 2019-11-07

**Authors:** MT Alrefai, CL Tarola, R Raagas, K Ridwan, M Shalal, N Lomis, A Paul, MD Alrefai, S Prakash, A Schwertani, D Shum-Tim

**Affiliations:** 1 Divisions of Cardiac Surgery and Cardiology, McGill University Health Center, Montreal, QC, Canada; 2 King Faisal Specialist Hospital and Research Center, Jeddah, Saudi Arabia; 3 Division of cardiac surgery, University of Western Ontario, Canada; 4 Division of Biomedical Engineering, McGill University Health Center, Montreal, QC, Canada; 5 Department of Chemical and Petroleum Engineering, Bioengineering Graduate Program, School of Engineering, University of Kansas, Lawrence, KS, USA; 6 Alnoor hospital, Ministry of health, Makkah, Saudi Arabia

## Abstract

**Objectives:**

Cell-based therapies have demonstrated variable degrees of success in the management of myocardial infarction and heart failure. By inducing a myocardial infarction in a rat model, the effects of secretome from human induced pluripotent stem cells (HiPSCs) and human mesenchymal stem cells (hMSCs) on cardiac function and remodeling were investigated.

**Methods:**

HiPSCs and hMSCs were cultured and after 12 cycles, secretome was collected. The quantification of stem cell growth factors was measured using the ELISA test. Thirty female Lewis rats underwent surgical ligation of the left coronary artery. The rats were then randomized (n=10/group) to receive one of three treatments injected into the peri-infarct area; normal saline, HiPSC and hMSC. Left ventricular ejection fraction (LVEF), fractional shortening (FS), histology and serum proteomics were evaluated in a blinded fashion both pre-operatively and at 2, 4 and 6 weeks.

**Results:**

ELISA studies revealed, Platelet-derived growth factor (PDGF) concentration of 3.35± 0.031 ng/ml (0.68± 0.027ng/ml) for MSC-CM group, 3.44± 0.042 ng/ml (0.78± 0.03 ng/ml) for the HiPSC-CM group, 3.2± 0.107 ng/ml (0.64±0.013 ng/ml) for the MSC-pre-group, 3.1± 0.075 ng/ml (0.71± 0.013 ng/ml) for the HiPSC-pre group and 3.3± 0.047 ng/ml (0.71± 0.014ng/ml) for the HiPSC-pre-r group at 60 min in comparison to at (0 min).

Compared to non-treated (NT), HiPSC and hMSC, treated rats demonstrated significant improvement in LVEF and FS, and significant reduction in scar size (p<0.05) at 4 and 6 weeks. Proteomic analysis detected the presence of Vascular endothelial growth factor (VEGF) in the serum of rats receiving HiPSC, which was absent in the NT and hMSC groups.

**Conclusion:**

The current study demonstrated a significant improvement of cardiac function and remodeling in response to secretome from HiPSCs and hMSCs. These findings suggest that secretome from HiPSCs may have potential therapy for acute myocardial infarction (MI) without the need of stem cell harvesting and implantation.

## Introduction

MI is contributing to global morbidity and mortality associated with cardiovascular disease^1^. Subsequent to a myocardial infarction (MI), contractile cardiomyocytes become necrotic and are replaced by non-contractile fibroblasts and collagen-rich scar tissue, resulting in a thin ventricular wall, decreased ejection fraction, and congestive heart failure (CHF) [1]. Although some evidence exists demonstrating age-dependent cardiomyocyte annual turnover between 0.45% - 1% and a limited regenerative capacity following MI, this response compared to inflammation is clinically insignificant [2,3]. Several investigations have demonstrated cardiomyocyte mitotic indices of 0.015 to 0.08% in CHF and post-infarct specimens, challenging that the heart is a post-mitotic organ and suggesting there may be a myocyte subpopulation that remains undifferentiated [3–5]. However, it is unclear what is the source of this regenerated cardiomyocytes, and if they are derived from progenitor cells or from the native cardiomyocytes. Moreover, it has been described that bone marrow-derived stem cells have homing ability to migrate toward the injured myocardium with the capacity to differentiate into cardiomyocyte like cells [6]. This present study is a continuation of previous studies done on stem cell- inspired secretome-rich injectable hydrogels for cardiac tissue repair [7,8].

Recent interest in using stem cells to enhance cardiomyocyte regeneration and ventricular remodeling in post-MI patients has led to investigation of various autologous cell lines including bone-marrow derived stem cells, resident cardiac stem cells, skeletal myoblasts, and adipose derived stem cells, which reduce the risk of allogenic rejection. These cell lines have been administered transendocardially, transepicardially, and via the coronary arteries in both human and animal models with varying degrees of success [9–11]. The ideal source of human cardiomyocyte progenitors has yet to be identified. However, a further understanding of the differentiation of embryonic stem cells and HiPSCs into cardiomyocytes will address this concern [12,13]. Moreover, adverse events including arrhythmias and teratoma/teratocarcinoma formation using stem cell grafting techniques, may hinder this methodology [14,15].

The paracrine mechanism suggests that stem cells secrete multiple complementary cellular pathways, promoting different cellular functions including anti-apoptosis, angiogenesis, and attenuation of fibrosis. Previous investigations have endeavored to exploit this mechanism using cell lysates/extracts to improve cardiac function and angiogenesis post-MI [16–18].

The aim of this study is to investigate the paracrine effects of stem cells on post-MI functional recovery and scar size in an ischemic rat heart model. It has been hypothesized that signaling molecules such as vascular endothelial growth factor (VEGF) and platelets derived growth factor (PDGF) present in cell secretome would have angiogenesis and neovascularization properties to improve myocardial function and reduction in scar size post MI. Bone marrow derived hMSCs and subcutaneous tissue reprogrammed cells were the stem cells used to produce HiPSCs. The main signaling molecules VEGF and PDGF, which primarily drive neovascularization and myocardial recovery, were focused on within this study.

## Methods

### Cell culture

Human bone marrow-derived hMSC’s were donated by Dr. Yen BL at the Institute of Cellular and System Medicine (Zhunan, Taiwan). The hMSC’s were isolated using previously described protocols [19].

HiPSCs isolated from fibroblasts within the skin of normal tissue donors and reprogrammed by episomal plasmid retroviral expression of OCT4, SOX2, KLF4 and MYV genes, were obtained from ATCC (Manassas, VA) along with all cell culture reagents. The cells were feeder-free (Pluripotent Stem Cell SFM XF/FF), and a biological matrix (cell matrix basement gel) was used in place of fibroblast feeders to provide a surface for attachment of the hiPSCs. Cells were cultured in T75 flasks in DMEM: F-12 medium. Culture medium (CM) was changed every 48 hours. Cells at 80–90% confluence were split or harvested. To split or harvest cells, the CM was aspirated, and cells were rinsed twice with 4 mL of Dulbecco’s-PBS (D-PBS). Stem cell dissociation reagent, 2 mL of 0.25% Trypsin was added and then followed by 10 to 15 minutes of incubation at 37°C in a humidified incubator at 5% CO_2_ and 21% O_2_. After that, 2 mL of DMEM: F-12 with ROCK inhibitor Y27632 was added to detach the cells. Suspended cell aggregates were then centrifuged at 200xg for 5 minutes at room temperature and cells were again re suspended in 1 mL of DMEM: F12 + ROCK inhibitor, and re-plated with a total of 10 mL of CM.

### Sample collection

CM from hMSC’s and HiPSC’s was collected for subsequent analysis prior to cell splitting when cells have reached 80–90% confluence rate. CM was collected and filtered using an Amicon® Ultra-15 Centrifugal Filter (MW 10 kDa, Merck Millipore, Billerica, MA). The aspirate secretome was centrifuged at 14000 rpm for 10 minutes and stored at −80°C. This was completed for 12 consecutive cycles.

### Measurement of VEGF and PDGF

An ELISA kit (Peprotech, rocky hill, NJ) was used to estimate the quantities of VEGF and PDGF present in each cell line’s secretome. ELISA microtiter plates were pre-coated with a murine monoclonal antibody against the media cytokine (VEGF or PDGF) being measured, following the manufacturer’s instructions. The absorbance was measured at 405 nm with wavelength correction at 650 nm within a period of 60 minutes at 15 minutes intervals.

### Animal study

The female Lewis rats selected were between 200–250 g in weight (Charles River Laboratories, Senneville, Canada). All animal studies were performed in accordance with the guidelines set forth by the Canadian Council on Animal Care and were approved by the institutional ethics committee.

The rats were anesthetized, intubated, and mechanically ventilated at 85 breaths/minute. The left coronary artery was accessed via a left thoracotomy through the fourth intercostal space and permanently ligated 2 mm from its origin with a 7/0 polypropylene suture(Ethicon Inc, Somerville, NJ). The ischemic myocardial segment rapidly became identifiable by observing pallor and akinesia corresponding to the distribution area of the left coronary artery. Fifteen minutes after ligation of the artery, 3 equal peri-infarct intramyocardial injections, totaling 500 μL, of the previously harvested MSC or HiPSC culture media or normal saline were completed using a 27-gauge needle. The experimented animals survived up to 8 weeks for analysis. The rats were randomized into three groups: (i) non-treated group (n = 10, normal saline), (ii) treatment group HiPSC (n = 10, HiPSC secretome), (iii) treatment group hMSC (n = 10, hMSC secretome). All groups had coronary ligation and injection of treatment into the peri-infarct area.

### Echocardiography

Trans-thoracic echocardiographic examinations were performed under inhaled isoflurane anesthesia (2.5% in oxygen, 500–700 mL/minute). Each rat had an echocardiography before surgery, to ensure baseline measurement, then immediately after ligation and at 1, 2, 4 and 6 weeks. These images were obtained using a commercially available system (Micromaxx P04224; SonoSite, Bothell, WA).

Measurements were recorded slightly inferior to the apex of the papillary muscles of the mitral valve. The time of end-diastole was defined as time of maximum diameter of the LV in one heart cycle and end-systole was defined as the minimum diameter. Following the “leading-edge” method, two images, on average, were obtained in each view and averaged over three consecutive cycles.

### Scar area analysis

To analyze the effect of treatment, a scar area analysis was performed. After cardiac excision, the hearts were immediately immersed in saline to remove excess blood from the ventricles. Samples were fixed in neutral-buffered 4% formalin and paraffin. Paraffin-embedded samples were sectioned at 5 μm. Masson’s trichrome staining (DBS, Pleasanton, CA) was performed to delineate scar tissue (blue color) from the total area of normal myocardium. Masson’s trichrome-stained sections were captured as digital images and analyzed using Image-J software (version 1.41; National Institutes of Health, Bethesda, MD) and Image Scope software (© 2016 Leica Biosystems Imaging). Infarct areas were calculated and expressed as a percentage. Samples were obtained at time points 1, 2, 4, 6, and 8 weeks. The results fromweeks 2, 4, 6 and 8 were combined to increase the sample size for analysis.

### Assessment of angiogenesis

Neovascularization was evaluated by analyzing the capillary and arteriole density in the peri-infarct zone. Immunofluorostaining was performed by using antibodies against CD31 (Santa Cruz Biotechnology Inc, Santa Cruz, CA) to identify capillary endothelial cells. To measure capillary density, three fields in the peri-infarct area were imaged, and the number of capillaries with diameter of 10|im or greater were counted. The capillary density (mean total CD31- positive microvessels)/mm^2^) was quantified using the average of three tissue sections spanning the peri-infarct tissue region of each heart.

### Proteomics analysis

We used the rat cytokines array panel A (R &D system, Inc., Minneapolis, MN) according to the manufacturer instructions to determine the concentration of soluble cytokines in the rat’s serum. This kit allows us to measure different cytokines simultaneously, such as VEGF, fractalkine, Interleukin-3, Interferon gamma, soluble intercellular adhesion molecule −1 (SICAM-1) *andL-selectin (CD62L).*

### Statistical analysis

Values are expressed as mean±SD and/or mean ± SEM. Typically, groups were tested and performed in triplicates and were compared with the student’s t-test, or by using the multivariant analysis of variance (MANOVA) test. Results were considered significant if the *p*-value was < 0.05.

## Results

### *In-vitro* results

Our analysis quantified VEGF and PDGF produced by *in-vitro* hMSCs and HiPSCs. There were 5 groups: “MSC-CM” and “HiPSC- CM” were the conditioned media harvested from cultured MSCs and HiPSCs, respectively. While “MSC-pre” was the MSC conditioning media before cell culture (DMEM), “HiPSC-pre” was HiPSC culture media before cell culture (DMEM: F12-no rock), “HiPSC-pre-r” was HiPSC conditioning media before cell culture with *rock inhibitor* (DMEM: F12-with rock).

There was a significant increase in VEGF and PDGF concentrations between each cell line and its control at 0 through 60 minutes, as shown in (Table 1). Additionally, there was a significant increase in the release of both growth factors in the HiPSC culture media compared to the MSC media at 60 minutes (PDGF: P= 0.022; VEGF: P= 0.0016).

ELISA studies reveled PDGF concentration of 3.35±(0.031) ng/ml for MSC-CM group, 3.44±(0.042) ng/ml for HiPSC-CM group, 3.2±(0.107) ng/ml for MSC-pre-group, 3.1±(0.075) ng/ml for HiPSC-pre group and 3.3±(0.047) ng/ml for HiPSC-pre-r group at 60 min in comparison to 0.68±(0.027) ng/ml for MSC-CM group, 0.78±(0.03) ng/ml for HiPSC-CM group,0.64±(0.013) ng/ml for MSC-pre-group, 0.71±(0.013) ng/ml for HiPSC-pre group and 0.71±(0.014) ng/ml for HiPSC-pre-r group at 0 min. It also showed, a VEGF concentration of 3.3±(0.051) ng/ml, 3.4±(0.024) ng/ml, 3.3±(0.03) ng/ml, 3±(0.029) ng/ml and 3±(0.017) ng/ml for MSC-CM group, HiPSC-CM group, MSC-pre group, HiPSC-pre group and HiPSC- pre-r group respectively at 60 minutes compared to1.07± (0.020) ng/ ml, 1.1± (0.017) ng/ml, 1.01±(0.029) ng/ml, 1.012±(0.009) ng/ml and 1.014±(0.013) ng/ml for MSC-CM group, HiPSC-CM group, MSC-pre group, HiPSC-pre group and HiPSC-pre-r group respectively at 0 minutes.

### *In vivo* study

**Figure 1:**
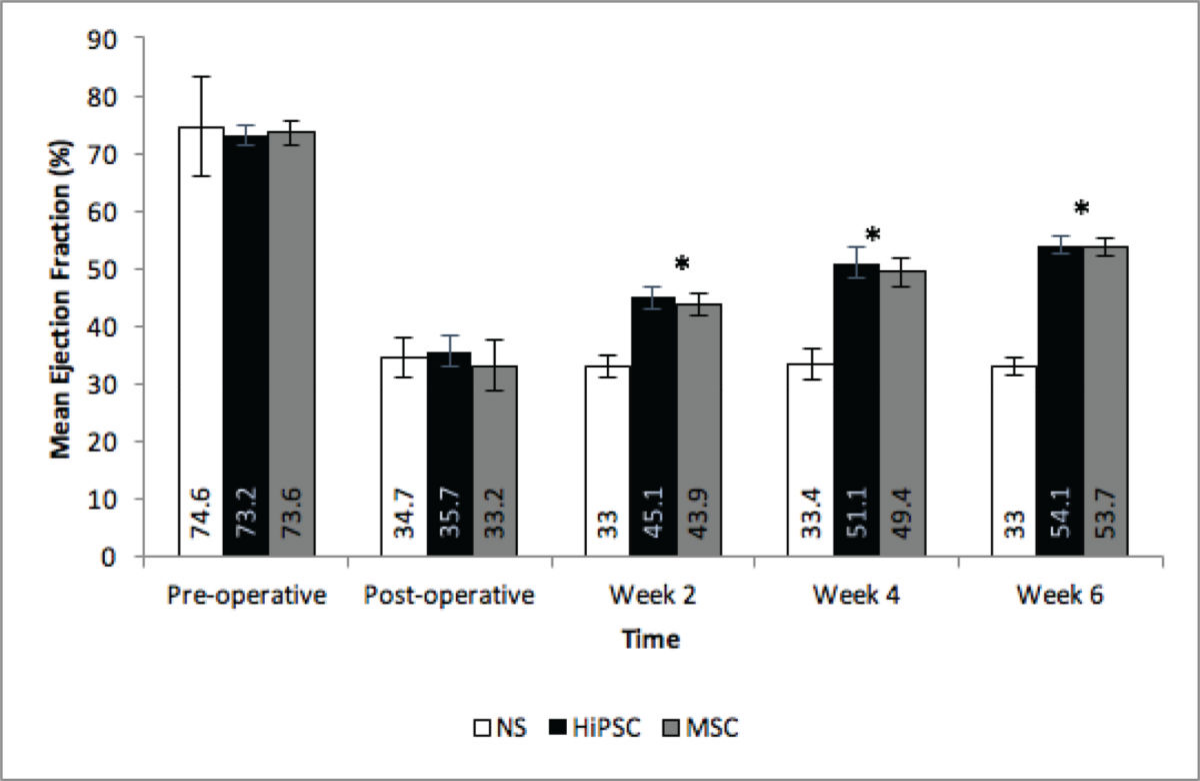
Effect of secretome treatment on Cardiac Function as EF. Change in mean EF over time across treatment groups. NT = Non-treated group; MSC = Mesenchymal Stem Cell Conditioned Media Group; HiPSC = Human Induced Pluripotent Stem Cells Conditioned Media Group * Indicates significant difference between both MSC and HiPSC compared to control.

**Figure 2:**
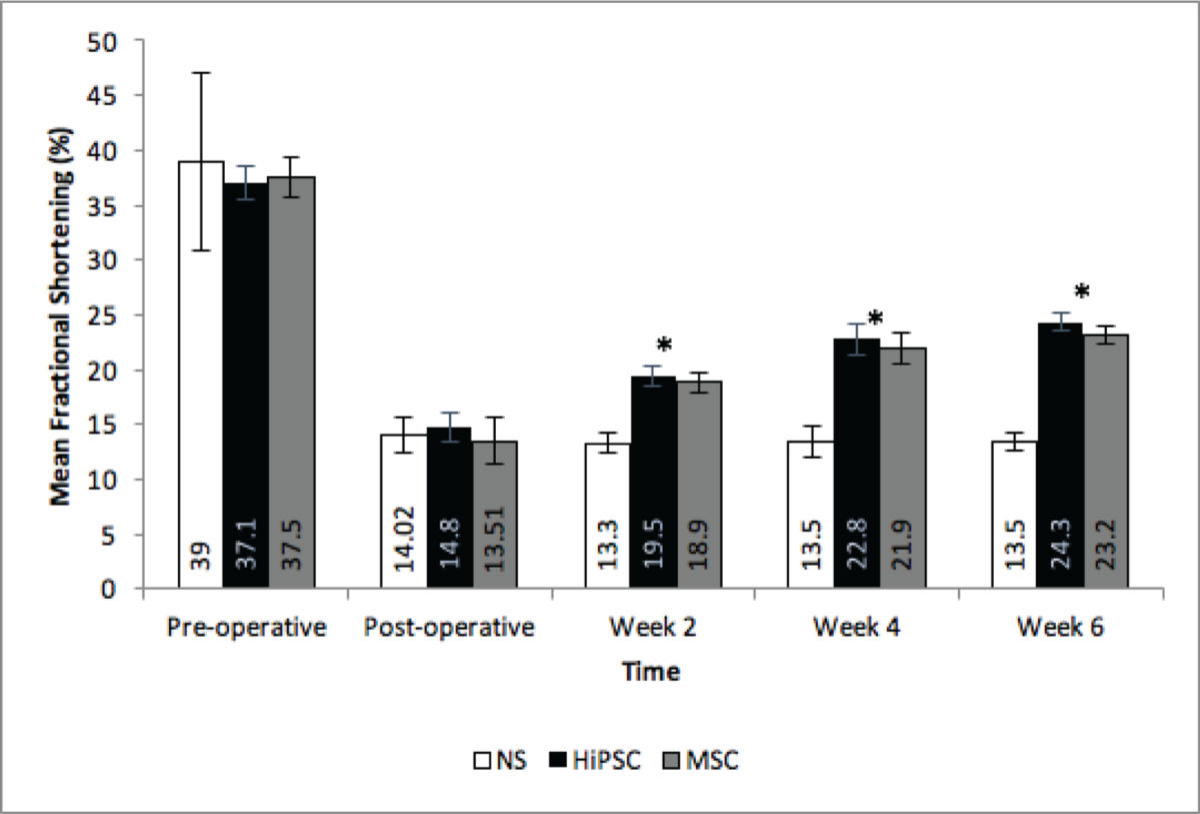
Effect of secretome treatment on Cardiac Function as FS. Change in mean FS over time across treatment groups. * Indicates significant difference between both hMSC and HiPS Ccompared to NT. NT =Non-Treated Group; hMSC= Mesenchymal Stem Cell Secretome Group; HiPSC= Human Induced Pluripotent Stem Cells Secretome Group

The animals were separated into 3 groups: NT, HiPSC and hMSC. Pre-operative echocardiography demonstrated no significant difference in LVEF or FS between the three groups (Table 2). Additionally, there was no significant difference between pre-operative LVEF or FS between the two treatment groups, where LVEF for Group HiPSC and Group hMSC (73± 2.4 vs. 73± 2.7, respectively), and FS for group HiPSC and Group hMSC (37.3±2vs. 37.4 ± 2, respectively). Following surgery, there was a rapid deterioration in both LVEF and FS means, in all groups, when compared to the pre-operative measurements (Table 2). There was no significant difference in mean LVEF or FS between groups immediately following surgery *(LVEF,* NT vs. HiPSC: (34.7±3.4 vs. 35.7±2.6), NT vs. hMSC: (34.7±3.4 vs. 33.1± 4.4), hMSC vs. HiPSC: (35.7±2.6 vs. 33.1±4.4) p = 0.501; *FS,* NT vs. HiPSC: (14±1.6 vs. 14.8 ±1.3), NT vs. hMSC: (14 ±1.6 vs. 13.5±2), hMSC vs. HiPSC: (13.5±2 vs. 14.8±1.3). In contrast, there was a significant difference in LVEF between treatment and control groups as early as week 2 (42.7±8.1, P<0.0001) (Figure 1). Similarly, there was a significant difference in FS at weeks 2, 4, and 6 compared to baseline in both the HiPSC and hMSC groups (Figure 2). There was no significant difference in mean LVEF (2 weeks: P=0.169; 4 weeks: P=0.864; 6 weeks: P=0.942) or mean FS (2 weeks: P=0.166; 4 weeks: P=0.907; 6 weeks: P=0.893) between the two treatment groups at any pre-determined time point.

### Scar area analysis

**Figure 3:**
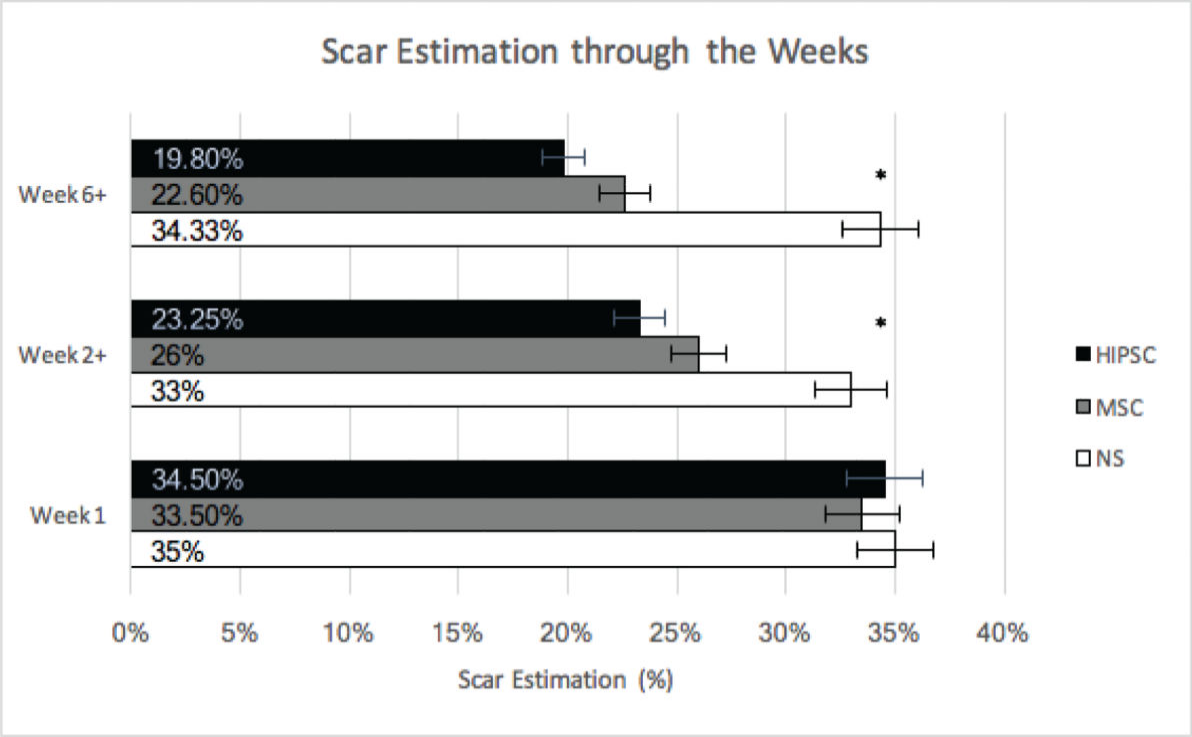
Effect of secretome treatment on fibrotic area. Estimated scar size post-operatively and following treatment at weeks 2 and 6. NT = Non-treated group; hMSC= Mesenchymal Stem cell Secretome Group; HiPSC = Human Induced Pluripotent Stem Cells Secretome Group. * Indicates significance between both MSC and HiPSC compared to control as well between HiPSC and MSC Groups.

**Figure 4:**
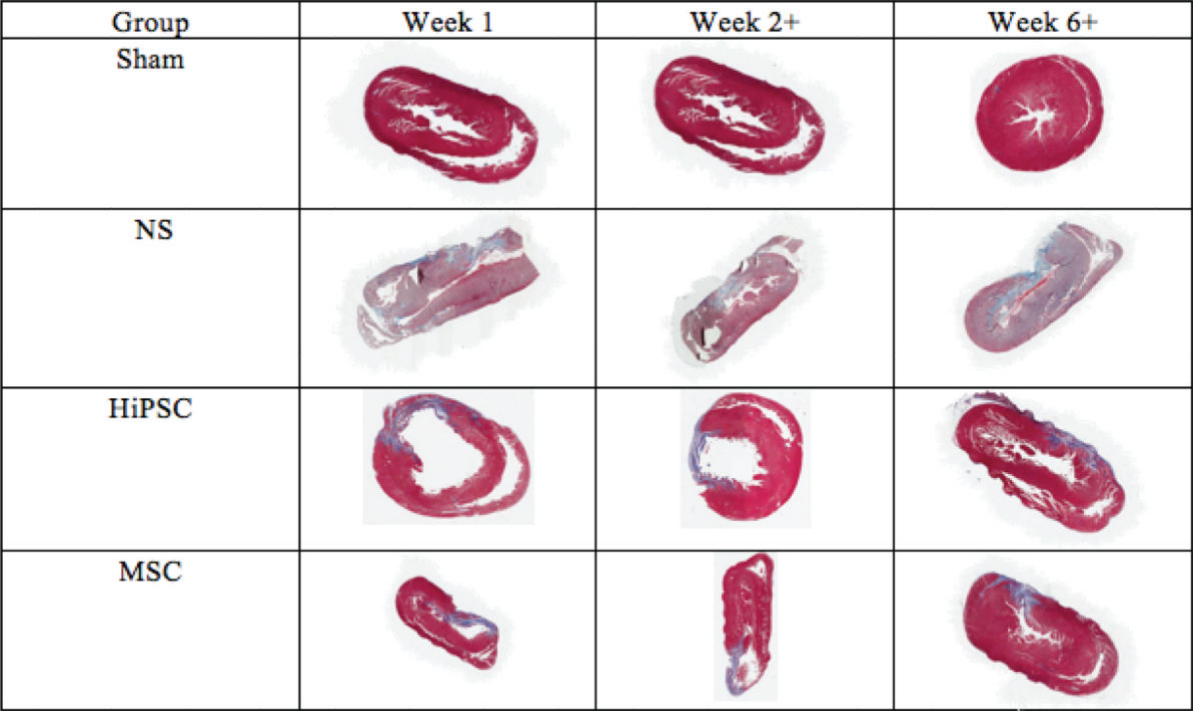
Effect of secretome treatment on Histological analysis. Histological scar area post-operatively and after treatment at weeks 2 and 6.

In the control (NT), hMSC, and HiPSC groups, hearts were harvested after the following time points, 1, 2, 4, 6 & 8 weeks post-surgery. The scar tissue area was quantified at weeks 1, 2+ (2–4 grouped) and 6+ (6–8 grouped). There was no statistically significant difference between all groups at week 1 (P=0.385). There was a statistically significant decrease in scar area in hMSC and HiPSC groups compared to the NT group (Figure 3) at weeks 2–4 (NT vs. hMSC: (33±0.01 vs. 26 ±0.01; P=0.001); NT vs. HiPSC: (33±0.01 vs. 23.25±0.009; P< 0.0001) and 6–8 (NT vs. hMSC: (34.3±0.011 vs. 22.6±0.016; P<0.0001); NT vs. HiPSC: (34.3±0.011 vs. 19.8±0.017; P<0.0001). Furthermore, at 2–4 weeks post-surgery, there was significant reduction in scar area in the hMSC group compared to the HiPSC group, with mean difference of 24.42 ±1.7, P=0.014. It was also evident at weeks 6–8, with mean difference of (21±2.1, P=0.024 (Figure 3). Histological demonstration of the reduction in scar size in response to treatment is shown in (Figure 4).

### Neovascularization and CD31 immunostaining

**Figure 5:**
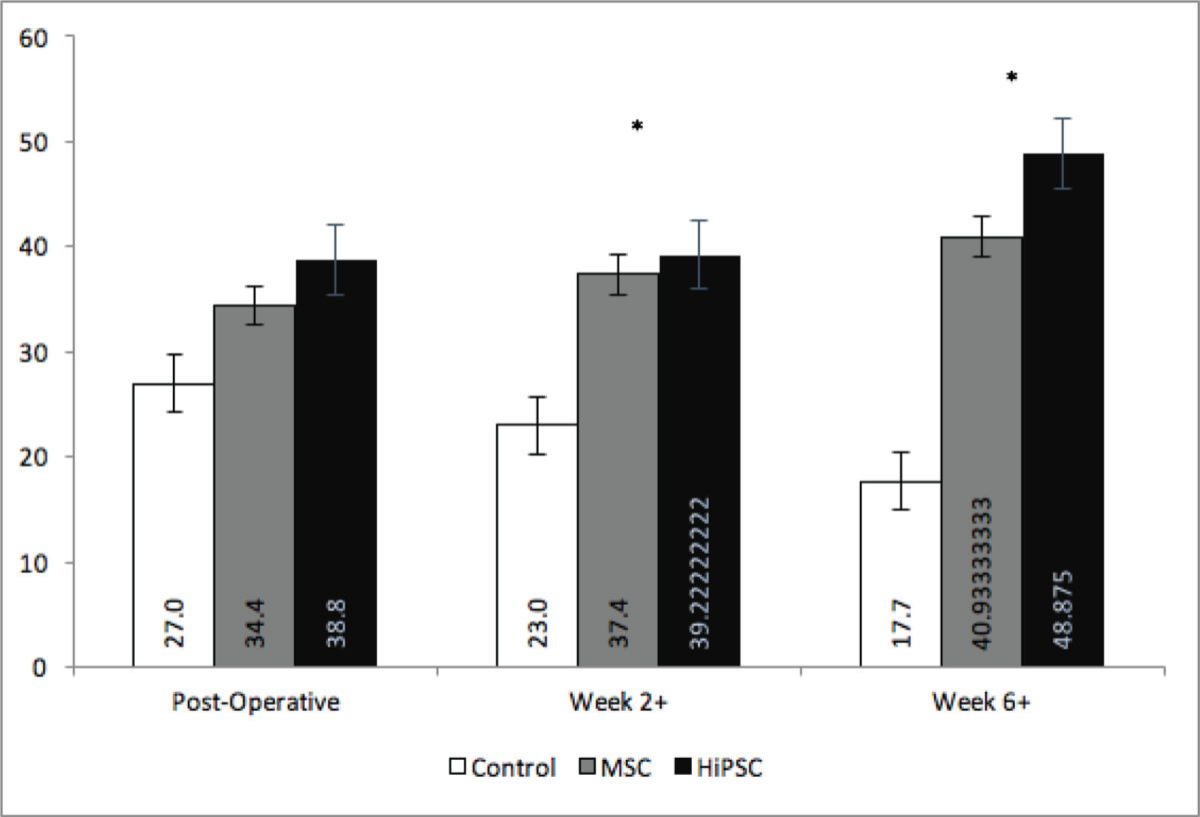
Effect of secretome treatment on Myocardial angiogenesis. CD31 immunostaining to identify peri-infarct endothelial cell density post-infarct. The vertical axis represents the cell numbers detected in high field under microscopy.

Immuno fluorescent staining was performed to assess CD31 staining in endothelial cells at the peri-infarct zone. Each group was again divided into 3 timeframes: week 1 (post-operative), week 2+ (weeks 2–4) and week 6+ (weeks 6–8) (figure 5). A significant increase in peri-infarct angiogenesis in the HiPSC and hMSC groups was found compared to controls at weeks 2+ (hMSC vs NT: (37.4±6.9 vs. 23±3.7) with mean difference of (29.5±9), P=0.003, HiPSC vs. NT: (39.2±11.5 vs. 23± 3.7) with mean difference of (32.7±12), P=0.006) and weeks 6+ (hMSC vs NT: (40.9 ± 14 vs. 17.6±8), with mean difference of (32±16), P<0.0001, HiPSC vs. NT: (48.8±11.4 vs. 17.6 ± 8) with mean difference of (37.6±18), P<0.0001). There was no significant difference in angiogenesis between the HiPSC and hMSC groups at weeks 2+ (39.2±11.5 vs. 37.4±6.9), with mean difference of (38.5±9.8), P=0.72 or 6+ (48.8±11.4 vs. 40.9 ±14), with mean difference of (45±13), P=0.097.The numbers here represented as cells/field.

### Proteomics analysis

**Figure 6:**
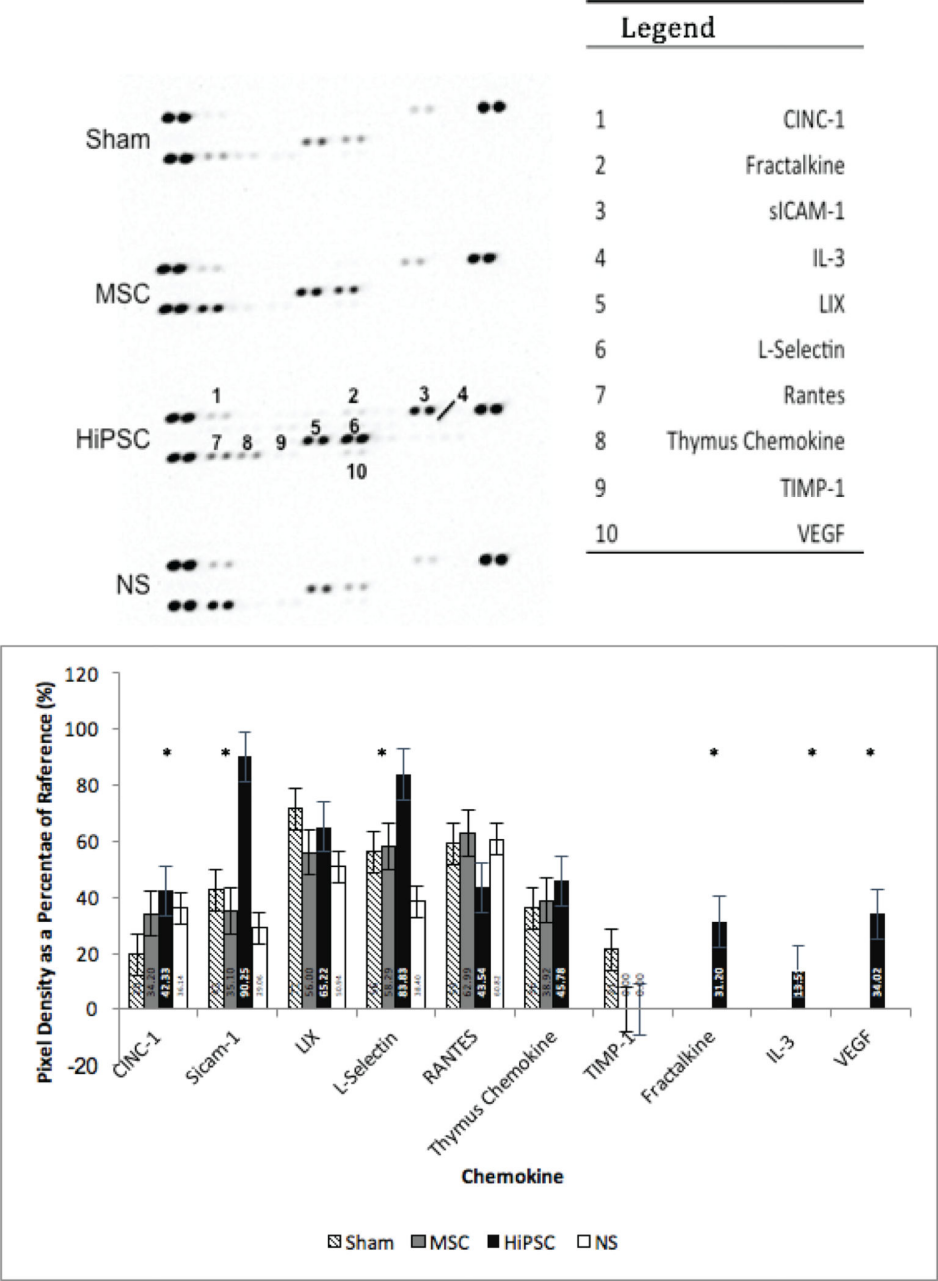
Effect of secretome treatment on Blood serum cytokine profile. Rat cytokine array for sham, control, and treatment group (MSC, HiPSC) rats. * Indicate high concentration and statistical significance.

The proteomics analysis (Figure 6) revealed abundant presence of VEGF, fractalkine and Interleukin-3 in the serum of group HiPSC that was absent in the NT and hMSC groups. Among the other factors detected in HiPSC group, a higher concentration of VEGF, SICAM- *1and L-select in (CD62L) were detected.*

## Discussion

Although the use of stem cell-based therapy has shown a promise in the treatment of MI and CHF, the benefits remains limited and associated with significant side effects [20,21]. In this present study, the potential role for cytokines to initiate myocardial repair in infarcted myocardial tissue was assessed, and a cell lineage capable of producing the cytokines VEGF and PDGF, was identified. The key findings were: i) both HiPSCs and hMSCs secrete VEGF and PDGF in an *in vitro* environment; ii) injection of the culture medium from both cell types containing these cytokines into peri-infarcted tissue resulted in improved LVEF and FS, post MI in the LAD territory; iii) stem cells secrete a number of other growth factors and chemokines that may improve cardiac function post-MI.

Based on the initial hypothesis of the paracrine effect of stem cells, the focus was on VEGF and PDGF given their direct relationship to angiogenesis and neovascularization [22–25]. Notably, both VEGF and PDGF also demonstrate some adverse effects. When administered in high intravenous doses, they could potentially lead to tumor pathogenesis and organ fibrosis [26,27]. However, both substrates were used in a localized fashion, with direct injection into the peri-infarct tissue. Measurable amounts of VEGF and PDGF were detected in the culture media postinjection of HiPSCs and hMSCs, which improved the myocardial remodeling properties associated with experimental models of MI.

The media from both stem cell lines were injected into the peri-infarct area rather than grafting the stem cells since previous investigations have demonstrated teratogenicity and arrhythmia disturbances with stem cell transplantation [15,28,29]. Overall, a reduction in scar area, increase in peri-infarct angiogenesis, and improved LVEF and FS using this technique was achieved. The results are also comparable to other investigations using stem cell grafting techniques which showed improved cardiac function following MI or ischemic cardiomyopathy [30].

The CD-31 immunostaining showed significantly more peri-infarct vascularization in hMSC or HiPSC culture media treated rats compared to the non-treated. In corroboration with echocardiographic results, these findings are consistent with those of Schuleri and colleagues, who identified post-infarct porcine hearts grafted with hMSCs had an increase in myocardial blood flow in infarcted tissue and suggested that this may reduce apoptosis and improve cardiac function [31]. This may explain the small scar size identified in rat hearts treated with hMSC and HiPSC culture medium at 6–8 weeks post-infarct. Although, the exact mechanism is unknown, itis hypothesized to be from the improvement of blood supply in the peri-infarct myocardium or possibly from the recruitment of cardiomyocyte progenitor cells.

A higher presence of VEGF, interleukin-3 (or multi-colony stimulating factor) and fractalkine was detected in rats treated with HiPSCs conditioned media. Several investigations have demonstrated the effect of these cytokines on cellular proliferation, differentiation, maturation, angiogenesis, and cardiac remodeling. Interleukin-3 has demonstrated improvement in left ventricular function and survival in animal models with acute MI or ischemic cardiomyopathy [32,33]. Fractalkine plays a role in delaying the enlargement of ventricular chambers following MI [34]. Higher concentrations of cytokine-induced neutrophil chemoattractant-1 (CINC-1), SICAM-1, and L-selectin were also detected in HiPSC culture media treated rats compared to the NT group. CINC-1 acts as a chemoattractant for polymorphonuclear neutrophils (PMNs) to the peri-infarct areas by interacting with cell surface chemokine receptors [35]. Finally, SICAM-1 and L-selectin have demonstrated roles as chemoattractant and in neutrophil function [36,37].

Since ischemic cardiomyopathy continues to remain a prominent cause of morbidity and mortality worldwide, investigation of stem cell therapy to improve cardiac function will continue. This stem-cell based therapy exploits the paracrine effect of stem cells to promote cardiac angiogenesis, reduce scar area, and improve cardiac function by minimizing some adverse effects associated with stem cell grafting. Translated to the clinical setting, these findings also suggest that secretome from hMSC and HiPSC can be prepared and made available on the shelf and potentially used in patients with acute MI and or ischemic cardiomyopathy. Therefore, it eliminates the logistics of highly specialized institutes and complex harvesting, proliferation and implantation processes while offering the benefits of stem cell-based therapy to the generalized population. Future studies aimed at characterizing the secretory profile of cell-based therapy may provide target specific treatment for patient with MI and CHF.

## Figures and Tables

**Table 1:** Comparison of VEGF and PDGF in the culture medium.

	0 min		60 min	
Comparison	Mean Difference* [SD]	P-value	Mean Difference* [SD]	P-value
**VEGF**				
MSC-CM VS HIPSC-CM	0.001 [0.02]	0.9611	0.173 [0.096]	0.0016**
MSC-CM VS MSC-PRE	0.382 [0.03]	0.0006**		
HIPSC-CM VS HIPSC-PRE	0.388 [0.01]	<0.0001**		
HIPSC-CM VSHIPSC-PRE-R	0.268 [0.01]	<0.0001**		
**PDGF**				
MSC-CM VS HIPSC-CM	0.83 [0.03]	0.0562	0.263 [0.157]	0.022**
MSC-CM VS MSC-PRE	0.013 [0.02]	0.6430		
HIPSC-CM VS HIPSC-PRE	0.069 [0.01]	0.0327**		
HIPSC-CM VSHIPSC-PRE-R	0.073 [0.01]	0.0223**		

* Mean difference represents mean difference in concentration between two samples (ng/mL)

** Statistically significant

**Table 2:** Comparison of pre-operative and post-operative changes.

	**Pre-operative Ejection Fraction**			**Pre-Operative Fractional Shortening**		
**Group**	**Mean(%)**	**SD**	**P-value**	**Mean(%)**	**SD**	**P-value**
NT	74.6	8.5		39.0	8.1	
HiPSC	73.2	1.7	0.928*	37.1	1.5	0.849*
MSC	73.6	2.1	0.967*	37.5	1.8	0.903*
	**Post-operative Ejection Fraction**			**Post-Operative Fractional Shortening**		
NT	34.7	3.5	<0.0001^+^	14.02	1.6	<0.0001^+^
HiPSC	35.7	2.7	<0.0001^+^	14.80	1.3	<0.0001^+^
MSC	33.2	4.5	<0.0001^+^	13.51	2.1	<0.0001^+^

* p-value identifies significant difference between treatment groups compared to control.

+ p-value identifies significant difference between pre- and post-operative measurements within the same group

## Abbreviations

ATCC: American Type Culture Collection
HRP: Horseradish Peroxidase
CINC-1: Cytokine Induced Neutrophil Chemo-Attractant
LVEDD: Left Ventricle End-Diastolic Diameters
LVESD: Left Ventricle End-Systolic Diameters
LVEDV: Left Ventricular End- Diastolic Volume
LVESV: Left Ventricular End-Systolic Volume
SICAM-1: Soluble Intercellular Adhesion Molecule-1
OCT-4: Octamer Binding Transcription Factor-4
SOX2: Sex Determining Region Y-Box 2
KLF4: Kruppel Like Factor-4

## References

[1] Lozano R, Naghavi M, Foreman K, Lim S, Shibuya K, Aboyans V, (2012) Global and regional mortality from 235 causes of death for 20 age groups in 1990 and 2010: a systematic analysis for the Global Burden of Disease Study 2010. Lancet, 380(9859): 2095–2128. 23245604 10.1016/S0140-6736(12)61728-0 PMC10790329

[2] Bergmann O, Bhardwaj RD., Bernard S, Zdunek S, Barnabé-Heider F, Walsh S, (2009) Evidence for cardiomyocyte renewal in humans. Science, 324(5923): 98–102. 19342590 10.1126/science.1164680 PMC2991140

[3] Beltrami AP., Urbanek K, Kajstura J, Yan SM, Finato N, Bussani R, (2001) Evidence that human cardiac myocytes divide after myocardial infarction. N Engl J Med, 344(23): 1750–1757. 11396441 10.1056/NEJM200106073442303

[4] Kajstura J, Leri A, Finato N, Di Loreto C, Beltrami CA., Anversa P (1998) Myocyte proliferation in end-stage cardiac failure in humans. Proc Natl Acad Sci U S A, 95(15): 8801–8805. 9671759 10.1073/pnas.95.15.8801 PMC21157

[5] Meckert PC., Rivello HG, Vigliano C, Gonzalez P, Favaloro R, Laguens R (2005) Endomitosis and polyploidization of myocardial cells in the periphery of human acute myocardial infarction. Cardiovasc Res, 67(1): 116–123. 15949475 10.1016/j.cardiores.2005.02.017

[6] Orlic D, Kajstura J, Chimenti S, Jakoniuk I, Anderson SM., Li B, (2001) Bone marrow cells regenerate infarcted myocardium. Nature, 410(6829): 701–705. 11287958 10.1038/35070587

[7] Waters R, Pacelli S, Maloney R, Medhi I, Ahmed RP., Paul A (2016) Stem cell secretome-rich nanoclay hydrogel: a dual action therapy for cardiovascular regeneration. Nanoscale, 8(14): 7371–7376. 26876936 10.1039/c5nr07806g PMC4863075

[8] Paul A, Hasan A, Kindi HA., Gaharwar AK, Rao VT, Nikkhah M, (2014) Injectable graphene oxide/hydrogel-based angiogenic gene delivery system for vasculogenesis and cardiac repair. ACS nano, 8(8): 8050–8062. 24988275 10.1021/nn5020787 PMC4148162

[9] Traverse JH., Henry TD, Pepine CJ, Willerson JT, Zhao DX, Ellis SG, (2012) Effect of the use and timing of bone marrow mononuclear cell delivery on left ventricular function after acute myocardial infarction: the TIME randomized trial. Jama, 308(22): 2380–2389. 23129008 10.1001/jama.2012.28726 PMC3652242

[10] Sürder D, Manka R, Lo Cicero V, Moccetti T, Rufibach K, Soncin S, (2013) Intracoronary injection of bone marrow-derived mononuclear cells early or late after acute myocardial infarction: effects on global left ventricular function. Circulation, 127(19): 1968–1979. 23596006 10.1161/CIRCULATIONAHA.112.001035

[11] Perin EC., Willerson JT, Pepine CJ, Henry TD, Ellis SG, Zhao DX, (2012) Effect of transendocardial delivery of autologous bone marrow mononuclear cells on functional capacity, left ventricular function, and perfusion in chronic heart failure: the FOCUS-CCTRN trial. JAMA, 307(16): 1717–1726. 22447880 10.1001/jama.2012.418 PMC3600947

[12] Caspi O, Huber I, Kehat I, Habib M, Arbel G, Gepstein A, (2007) Transplantation of human embryonic stem cell-derived cardiomyocytes improves myocardial performance in infarcted rat hearts. J Am Coll Cardiol, 50(19): 1884–1893. 17980256 10.1016/j.jacc.2007.07.054

[13] Laflamme MA., Chen KY, Naumova AV, Muskheli V, Fugate JA, Dupras SK, (2007) Cardiomyocytes derived from human embryonic stem cells in pro-survival factors enhance function of infarcted rat hearts. Nat Biotechnol, 25(9): 1015–1024. 17721512 10.1038/nbt1327

[14] Kawamura M, Miyagawa S, Miki K, Saito A, Fukushima S, Higuchi T, (2012) Feasibility, safety, and therapeutic efficacy of human induced pluripotent stem cell-derived cardiomyocyte sheets in a porcine ischemic cardiomyopathy model. Circulation, 11: 126(11 Suppl 1). 10.1161/CIRCULATIONAHA.111.084343 22965990

[15] Lee AS., Tang C, Cao F, Xie X, van der Bogt K, Hwang A, (2009) Effects of cell number on teratoma formation by human embryonic stem cells. Cell cycle, 8(16): 2608–2612. 19597339 10.4161/cc.8.16.9353 PMC2866168

[16] Barile L, Lionetti V, Cervio E, Matteucci M, Gherghiceanu M, Popescu LM., (2014) Extracellular vesicles from human cardiac progenitor cells inhibit cardiomyocyte apoptosis and improve cardiac function after myocardial infarction. Cardiovasc Res, 103(4): 530–541. 25016614 10.1093/cvr/cvu167

[17] Leroyer AS., Ebrahimian TG, Cochain C, Récalde A, Blanc-Brude O, Mees B, (2009) Microparticles from ischemic muscle promotes postnatal vasculogenesis. Circulation, 119(21): 2808–2817. 19451354 10.1161/CIRCULATIONAHA.108.816710

[18] Yeghiazarians Y, Zhang Y, Prasad M, Shih H, Saini SA., Takagawa J, (2009) Injection of bone marrow cell extract into infarcted hearts results in functional improvement comparable to intact cell therapy. Molecular therapy. Mol Ther, 17(7): 1250–1256. 19384293 10.1038/mt.2009.85 PMC2835212

[19] Yen BL., Huang HI, Chien CC, Jui HY, Ko BS, Yao M, (2005) Isolation of multipotent cells from human term placenta. Stem cells, 23(1): 3–9. 15625118 10.1634/stemcells.2004-0098

[20] Pacelli S, Basu S, Whitlow J, Chakravarti A, Acosta F, Varshney A, (2017) Strategies to develop endogenous stem cell-recruiting bioactive materials for tissue repair and regeneration. Adv Drug Deliv Rev, 120: 50–70. 28734899 10.1016/j.addr.2017.07.011 PMC5705585

[21] Paul A, Ge Y, Prakash S, Shum-Tim D (2009) Microencapsulated stem cells for tissue repairing: implications in cell-based myocardial therapy. Regen Med, 4(5): 733–745. 19761398 10.2217/rme.09.43

[22] de Vries C, Escobedo JA., Ueno H, Houck K, Ferrara N, Williams LT (1992) The fms-like tyrosine kinase, a receptor for vascular endothelial growth factor. Science, 255(5047): 989–991. 1312256 10.1126/science.1312256

[23] Liu G, Li L, Huo D, Li Y, Wu Y, Zeng L, (2017) A VEGF delivery system targeting MI improves angiogenesis and cardiac function based on the tropism of MSCs and layer-by-layer self-assembly. Biomaterials, 127: 117–131. 28284103 10.1016/j.biomaterials.2017.03.001

[24] Wang Z, Kong D, Banerjee S, Li Y, Adsay NV., Abbruzzese J, (2007) Down-regulation of platelet-derived growth factor-D inhibits cell growth and angiogenesis through inactivation of Notch-1 and nuclear factor-kappaB signaling. Cancer research, 67(23): 11377–11385. 10.1158/0008-5472.CAN-07-2803 18056465

[25] Zhao W, Zhao T, Huang V, Chen Y, Ahokas RA., Sun Y (2011) Platelet-derived growth factor involvement in myocardial remodeling following infarction. J Mol Cell Cardiol, 51(5): 830–838. 21767547 10.1016/j.yjmcc.2011.06.023 PMC3628689

[26] Bonner JC. (2004) Regulation of PDGF and its receptors in fibrotic diseases. Cytokine Growth Factor Rev, 15(4): 255–273. 15207816 10.1016/j.cytogfr.2004.03.006

[27] Epstein SE., Kornowski R, Fuchs S, Dvorak HF (2001) Angiogenesis therapy: amidst the hype, the neglected potential for serious side effects. Circulation, 104(1): 115–119. 11435348 10.1161/01.cir.104.1.115

[28] Hentze H, Soong PL., Wang ST, Phillips BW, Putti TC, Dunn NR (2009) Teratoma formation by human embryonic stem cells: evaluation of essential parameters for future safety studies. Stem Cell Res, 2(3): 198–210. 19393593 10.1016/j.scr.2009.02.002

[29] Menasche P, Alfieri O, Janssens S, McKenna W, Reichenspurner H, Trinquart L, (2008) The Myoblast Autologous Grafting in Ischemic Cardiomyopathy (MAGIC) trial: first randomized placebocontrolled study of myoblast trans- plantation. Circulation, 117(9): 1189–1200. 18285565 10.1161/CIRCULATIONAHA.107.734103

[30] Ong SG., Huber BC, Lee WH, Kodo K, Ebert AD, Ma Y, (2015) Microfluidic Single-Cell Analysis of Transplanted Human Induced Pluripotent Stem Cell-Derived Cardiomyocytes After Acute Myocardial Infarction. Circulation, 132(8): 762–771. 26304668 10.1161/CIRCULATIONAHA.114.015231 PMC4557214

[31] Schuleri KH., Amado LC, Boyle AJ, Centola M, Saliaris AP, Gutman MR, (2008) Early improvement in cardiac tissue perfusion due to mesenchymal stem cells. Am J Physiol Heart Circ Physiol, 294(5): H2002–2011. 10.1152/ajpheart.00762.2007 18310523

[32] Chandrasekar B, Smith JB., Freeman GL (2001) Ischemia- reperfusion of rat myocardium activates nuclear factor-KappaB and induces neutrophil infiltration via lipopolysaccharide-induced CXC chemokine. Circulation, 103(18): 2296–2302. 11342480 10.1161/01.cir.103.18.2296

[33] Kobayashi M, Van Leeuwen BH., Elsbury S, Martinson ME, Young IG, Hapel AJ (1989) Interleukin-3 is significantly more effective than other colony-stimulating factors in long-term maintenance of human bone marrow-derived colony-forming cells in vitro. Blood, 73(7): 1836–1841. 2653465

[34] Xuan W, Liao Y, Chen B, Huang Q, Xu D, Liu Y, (2011) Detrimental effect of fractalkine on myocardial ischaemia and heart failure. Cardiovasc Res, 92(3): 385–393. 21840883 10.1093/cvr/cvr221

[35] Kim D, Haynes CL. (2012) Neutrophil chemotaxis within a competing gradient of chemoattractants. Anal Chem, 84(14): 6070–6078. 22816782 10.1021/ac3009548 PMC3404751

[36] Hafezi-Moghadam A, Thomas KL., Prorock AJ, Huo Y, Ley K (2001) L-selectin shedding regulates leukocyte recruitment. J Exp Med, 193(7): 863–872. 11283159 10.1084/jem.193.7.863 PMC2193368

[37] Sellak H, Franzini E, Hakim J, Pasquier C (1994) Reactive oxygen species rapidly increase endothelial ICAM-1 ability to bind neutrophils without detectable upregulation. Blood, 83(9): 2669–2677. 7513210

